# Seamless images stitching for 3D human models

**DOI:** 10.1186/s40064-016-3447-z

**Published:** 2016-10-10

**Authors:** Chao Lai, Fangzhao Li, Shiyao Jin

**Affiliations:** 1 National Laboratory for Parallel and Distributed Processing, National University of Defense Technology, Changsha, China; 2 College of Computer, National University of Defense Technology, Changsha, China

**Keywords:** Images stitching, Adaptive iterative factor, Smooth, Seamless

## Abstract

Realistic 3D human model reconstruction is an important component in computer graphics and computer vision. In particular, texturing on the surface of models is a key stage of reconstruction. In this paper, we dispose the texture mapping on the model’s surface as an optimization of image stitching, and present an effective method to generate a seamless, integrated and smooth texture on the surface of 3D human model. First, we build a corresponding Markov Random Field model with respect to color images and triangular meshes of the surface. On the basis of $\alpha$-expansion optimization for this Markov Random Field model, a 2D translation coordinate of color image, as an adaptive iterative factor, is introduced into the optimization to match the color content at the boundary of adjacent meshes. That compensates for the misalignment of adjacent color images, which caused by the inaccuracy of depth data and multi-view misregistration. Then we apply Poisson blending to a composite vector field in gradient domain, to resolve the small but noticeable illumination variations between different color images. To repair the blank regions, we parameterize the model’s surface and project it onto a 2D plane. Then the K-Nearest Neighbor algorithm is applied to fill up the blank regions with texture contents. Finally, we evaluate our method by comparison with another three advanced methods on some human models, and the results demonstrate that our method of images stitching creates a best texture on the surface of 3D human model both in visual effect and quantitative analysis.

## Background

Realistic 3D model reconstruction (Li et al. 2013) is an important component in computer graphics and computer vision, and comprehensively applied in our daily life, especially in films, animations, video games, virtual reality and so on. It is an underway revolution from 2D to 3D, which gradually makes the reconstruction of the real world come true. As an innovative technological application, 3D human reconstruction makes the humans get rid of the visual fatigue of 2D frames, and it opens a novel and fascinated domain to acquaint ourselves with the real world much more fascinating.

A high-quality texture on the surface of model is a key component of the reconstructed model, which impacts the visual effect significantly. In this paper, our goal is to generate a seamless texture on the surface of given 3D human model, from a set of images obtained by multiple RGB-Depth cameras.

In a large amount of previous works on this problem, they reconstruct an accurate 3D model and generate a preferable texture on the surface of model by using complex equipment, such as laser scanning and structured light. However, these methods require such an expensive and complex facility that can’t be used extensively.

The emergence of RGB-Depth cameras in recent years, such as Kinect produced by Microsoft and Xtion by Asus, paves the way to settle this matter. Evidently, it becomes widely applied due to its low price, simplified operation and hand-held shape, although the accuracy is not as high as the former.

Due to the limitation of accuracy of RGB-Depth camera, some dramatic misaligned seams are produced on the texture. But even using the facility of laser scanning and structured light, the inaccuracy of depth data and multi-view misregistration also result in these seams. Besides, some irrelevant contents of environment are mixed with the objective texture. In addition, the inaccuracy of depth data and multi-view misregistration result in some blank regions without any color contents, which destroy the integrity of texture. Furthermore, due to the different ray gains of cameras from respective directions, as well as surface reflection, the local variations in illumination is noticeable between color images, which damages the visual effect significantly and degrade the quality of texture.

In this paper, to eliminate these artifacts, we treat the texture mapping on model’s surface as an optimizaiton of images stitching, and propose a method of global optimization to compensate for the inaccuracy of depth data and multi-view misregistration. In our method, an extra adaptive iterative factor is introduced into the parameters of images stitching, and the texture images are allowed to move gradually to match the color content at the boundary of adjacent meshes. Finally, the misaligned seams are eliminated by iterative optimization.

Regarding irrelevant environmental contents, we build Gaussian Mixture Models (GMM) (Rother et al. 2004; Ruzon and Tomasi 2000; Chuang et al. 2001) in the color space, and segment the image into two parts, objective and environment. Therefore, only objective content remains in the images while the environmental contents are erased.

To solve the issue of illumination variations between adjacent color images, we construct a composite vector field in the gradient domain of color space, with the points located in the boundary as constraints. Due to the pixels distributing discretely, we solve the resulting discrete Poisson equation (Perez et al. 2003), and blend the adjacent color images smoothly. Thus the color of different images becomes coherent.

Last but not least, we parameterize the surface of model and project it onto a 2D plane. Then the K-Nearest Neighbor algorithm is applied to search for K points with color content, and the pixels in blank regions are filled with the average content of the K points. Then we back project the plane onto the surface, and all the blank regions become textured with color contents. Consequently, an integrated and consistent texture is generated on the surface of 3D human model, as shows in Fig. 6.

## Related work

It is a classic topic that creating a texture on the surface of objective model, and the key is to minimize the visible seams caused by multi-view misregistration and inaccuracy of data. Catmull (1974, 1980), begin to use the texture mapping skill to improve the visual quality of images generated by raster scanning facility. Rocchini et al. (1999) confirm the best texture for each triangular mesh by projecting the color images onto the surface of 3D model, and apply the region expansion algorithm to complete the ambient texture projection. It guarantees the validity of inner texture for local areas, but results in discontinuities in the boundary of images. Liu et al. (2006) resolve the discontinuities in the boundary effectively by global optimization of the multiple texture images using bundle adjustment. Zhou et al. (2005) implement coarse-to-fine strategy of several levels, considering both geometric and images in overlapping textures, and complete the textures montage.

Different from the previous work, it has become popular recently that projecting the model surface onto the color images and forming the texture as images stitching problem (Wang et al. 2001; Thormahlen and Seidel 2008; Dessein et al. 2014). It combines the images with triangular meshes as a Markov Random Field and constructs an objective energy function of cost. Despite the elegance and power of the energy minimization approach, the algorithms which were previously used, such as iterated conditional modes (ICM) (Besag 1993) and simulated annealing (SA) (Barnard 1989), proved to be ineffective. Boykov et al. (2001) present two algorithms based on graph cuts that effectively find a global minimum of the energy function. As is well known, it has been widely applied in a great amount of cases, such as stereo matching (Barnard 1989; Boykov et al. 2001), image segmentation (Rother et al. 2004; Boykov and Jolly 2001) and image denoising (Felzenszwalb and Huttenlocher 2006). Belief propagation (BP) (Felzenszwalb and Huttenlocher 2006; Yedidia et al. 2000; Tappen and Freeman 2003) is a powerful optimization algorithm to resolve the energy minimization and widely used in a large variety of tasks. Especially in stereo matching, it usually gets a dramatic result. However, BP is not guaranteed to converge and may go into an infinite loop switching between two labelings. If BP converges and there are no ties in the min-marginal for nodes, it often achieves the local minimum.

To eliminate the noticeable seams on the mosaic image, Lempitsky and Ivanov (2007) cast the problem of seams visibility minimization as an optimization of a mesh based Markov Random Field energy, and propose a seam leveling procedure to remove out the mosaic seams without affecting fine details of the texture. Without any move of the texture content, some distortions occur in the boundary of adjacent meshes, accompanied with some slight seams. Ran et al. (2010) present an automatic method to recover high-resolution texture over an object by mapping detailed photographs onto its surface, which minimizes the visible seams by applying the Tree-Reweighted Message Passing algorithm (TRW-s) (Vladimir 2006) to achieve a global minimization, and then assigns smooth texture to adjacent triangular meshes with a set of local image transformations. In practice, there is no guarantee that the energy might not actually increase with time, and the energy sometimes starts to oscillate.

 Soler et al. (2002) present an approach to form a seamless texture over the surface meshes by optimizing a set of per-face image transformations in a greedy manner. Eisemann et al. (2008) pose the method of optical flow to handle with these noticeable seams, which calculates the pairwise warping between the images and then blended at the rendering stage. Dessein et al. (2014) present a method to blend the sampled images by least-angle selection of gradients in overlapping patches, but ignore nonlinearity due to specularity and shadows. Dessein et al. (2015) segment the faces into patches, and blend the sampled textures to synthesise a seamless texture over the surface.

Among these methods, it is a common feature that each mesh of the surface can be project onto at least one image. However, in practice, it is general that blank regions without any texture content exist on the surface of model, caused by inaccuracy of depth data and multi-view misregistration. In this paper, we present a method of parameterization of the surface, combined with K-Nearest Neighbor algorithm, to deal with these blank regions, and then fill up them with color contents to create an integrated and coherent texture on the surface of model.

Color fusion in gradient domain (Fattal et al. 2002; Chuang et al. 2009) has been widely applied in a large variety of tasks to produce a new smooth image from the original images. Levin et al. (2004) apply gradient domain fusion in stitching the panoramic mosaics together. Raskar et al. (2004) blend multiple images of a common scene with different illumination intensity in the gradient domain, and consequently generate surrealist images with much more information. Perez et al. (2003) introduce a method of Poisson image editing to blend images into a single image by copying the objective region of a single image into a destination image in the manner of gradient domain. In our work, we add the points in the boundary of adjacent images into the Poisson equation as constraint to make it over-constrained.

In this paper, we propose a method of creating a seamless texture mapping on the model’s surface with a rapid optimization. We introduce an adaptive iterative factor to expand the texture, and the texture is allowed to move towards to match with adjacent meshes at the shared edge. Then the $\alpha$-expansion algorithm is applied to handle the energy optimization in an iterative strategy. The results of experiments demonstrate the effectiveness of our method both in visual observation and qualitative analysis.

## Seamless images stitching

### Texture generation

**Fig. 1:**
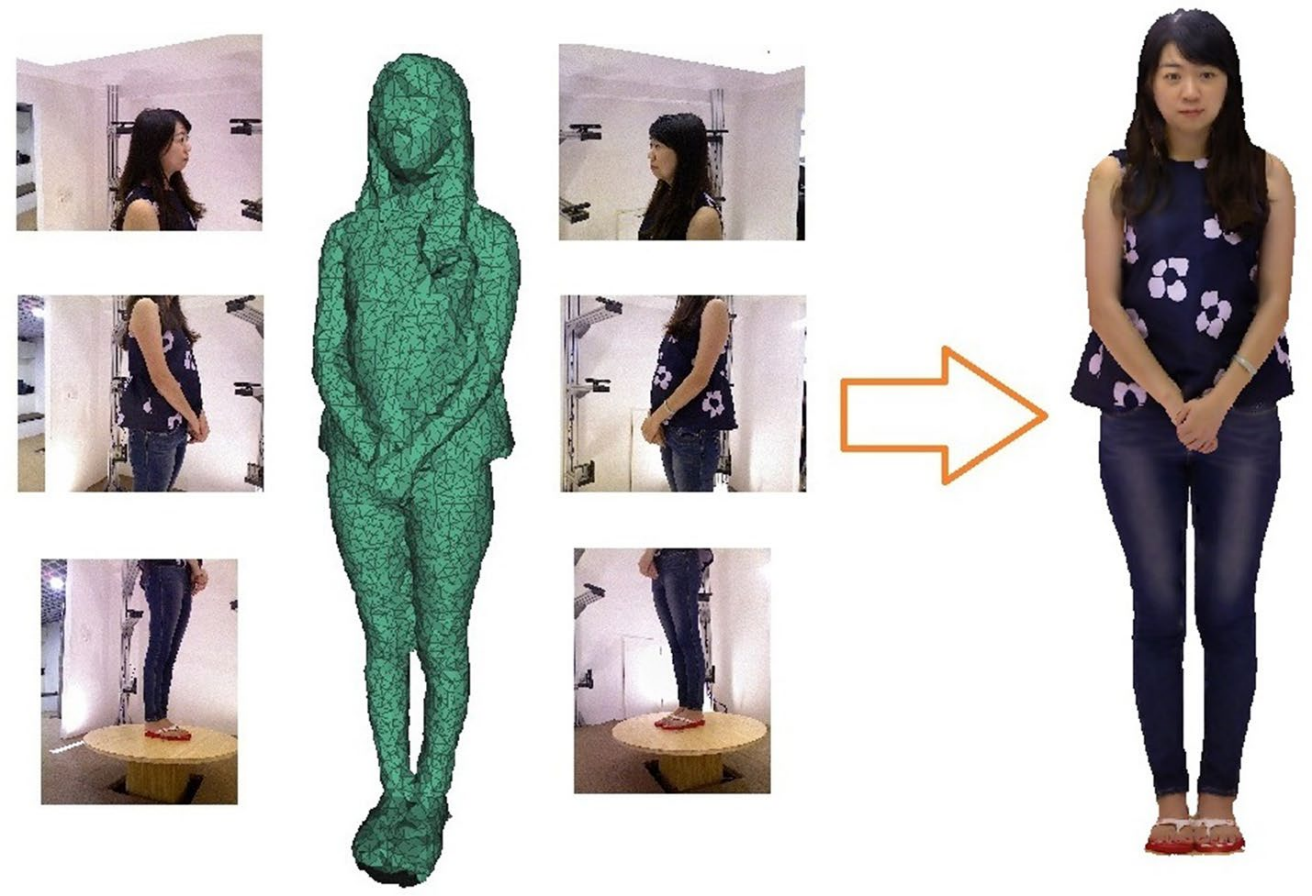
Overview of generating a texture on the surface of model. Given a set of images and a surface mesh of human model, we calculate a seamless texture for it. The images shown in this paper are generated by RGB-Depth cameras, and the surface mesh of human model is reconstructed previously by the point cloud of depth data

To cover the objective model, We set up multiple RGB-Depth cameras around it. Then a set of color images and depth data are simultaneously generated by these cameras, which are used to calculate a texture mapping between meshes of the surface and pixels in the color images. With the point cloud of depth data, we first reconstruct the mesh of model’s surface by the method which we proposed in paper (Lai et al. 2015). First we do the registration of the point cloud by the iterative closest point (ICP) algorithm (Besl and Mckay 1992). Then we optimize the noisy raw data by bilateral filtering algorithm (Christian et al. 2012) and finally apply the Poisson surface reconstruction algorithm (Kazhdan et al. 2006) to create the mesh of model’s surface. With the set of color images and the reconstructed mesh of model’s surface, we seek to reconstruct the texture from the images by using a projective map from each triangular mesh to one of the images, as shown in Fig. 1.

Once the color images and depth data are captured by the RGB-Depth cameras, we first register all of them into a global coordinate system, and calculate the 3D point’s position in the global coordinate system. The formula is

1 $$\begin{aligned} V = (d_n - \delta _d) \cdot q_d / \phi _d \end{aligned}$$

where *d* is the location in depth data, *q* is depth value, $\delta _d$ and $\phi _d$ are the center and projection parameters of depth data. Then the coordinate of 3D point in global coordinate system is obtained,

2 $$\begin{aligned} V' = R \cdot V + T \end{aligned}$$

where *R* is a rotation matrix and *T* is a translation vector. Therefore, the correspondent relationship between 3D points and pixels in the color images is established as follows,

3 $$\begin{aligned} p = V' \cdot \phi _c / q_d + \delta _c \end{aligned}$$

where $\delta _c$ and $\phi _c$ are the center and projection parameters of color images.

Consequently, the pixel is figured out for each point of the model, and then the texture is generated on the surface by projecting the color image content onto the model.

### Images enhancement

Due to the complex environment in the color images, as well as the inaccuracy of depth data, it is an obvious defect that some irrelevant environment contents exist in the final texture. So it is necessary to filter out the irrelevant environment (background), and keep the content of human model (foreground) alone in the color images. On the assumption that there are totally $P_n$ pixels in color image, we construct 2 Gaussian Mixture Models (GMM) in color space, one for foreground and the other for background. Each one is a full covariance Gaussian mixture with *T* components (typically $T = 5$). Therefore, a vector $t=\{t_1,\ldots ,t_n\},t_i\in \{1,\ldots ,T\}$, is formed to assign a unique GMM component to each pixel in the color image. For GMM components, we define $a = 1$ for foreground and $a = 0$ for background. Then the energy function for segmentation is introduced as follows,

4 $$\begin{aligned} E = E_d + E_s \end{aligned}$$

where $E_d$ is data penalty of pixels assigned to foreground or background, and it depends on the GMM components variables *t*. So $E_d$ is defined as follows,

5 $$\begin{aligned} E_d= & {} \sum D(a_n, t_n, p_n)\nonumber \\= & {} \sum \left( -\log P(p_n | a_n, t_n) - \log \delta (a_n, t_n) \right) \nonumber \\= & {} \sum \left( -\log \delta ( a_n, t_n) + \frac{1}{2} \log det \Sigma ( a_n, t_n)\right. \nonumber \\&\left. + \frac{1}{2} \left[ p_n - \mu (a_n, t_n) \right] ^{T} \Sigma (a_n, t_n)^{-1} \left[ p_n - \mu (a_n,t_n) \right] \right) \end{aligned}$$

where $P(\cdot )$ is a Gaussian probability distribution, $\delta$ is the mixture weighting coefficients, $\mu$ is the means and $\Sigma (a_n,t_n)$ is the covariance of the Gaussian components for the foreground and background distributions.

The second term $E_s$ is smooth energy of discontinuities between adjacent pixels assigned to foreground and background respectively, and calculated by Euclidean distance in the color space.

6 $$\begin{aligned} E_s = \gamma \sum _{(m,n)\in C}|a_n \ne a_m| exp \left( -\beta \left\| p_m - p_n \right\| ^{2} \right) \end{aligned}$$

For the energy minimization, we calculate it iteratively until convergence. First we set the whole image as the foreground $(a = 1)$, and initialize the foreground and background GMMs from sets $a = 1$ and $a = 0$ respectively. Then we perform the procedure of iterative minimization. Step 1, assign GMM components to pixels, which is straightforward and done by simple enumeration of the $t_n$ values for each pixel *n*. Step 2, it is implemented as a set of Gaussian parameter estimation procedures as follows. For a given GMM component *t* in the foreground model, the subset of pixels $F(t) = \{p_n : t_n = t , a_n = 1\}$ is defined. The mean $\mu (a,t)$ and covariance $\Sigma (a,t)$ are estimated in standard fashion as the sample mean and covariance of pixel values in *F*(*t*), and the weights are $\delta (a,t) = |F(t)| / \Sigma _t|F(t)|$, where |*F*(*t*)| denotes the size of *F*(*t*). Finally, step 3 is a global optimization, using minimum cut, exactly as this paper (Boykov and Jolly 2001). Repeat from step 1 until convergence.

**Fig. 2:**
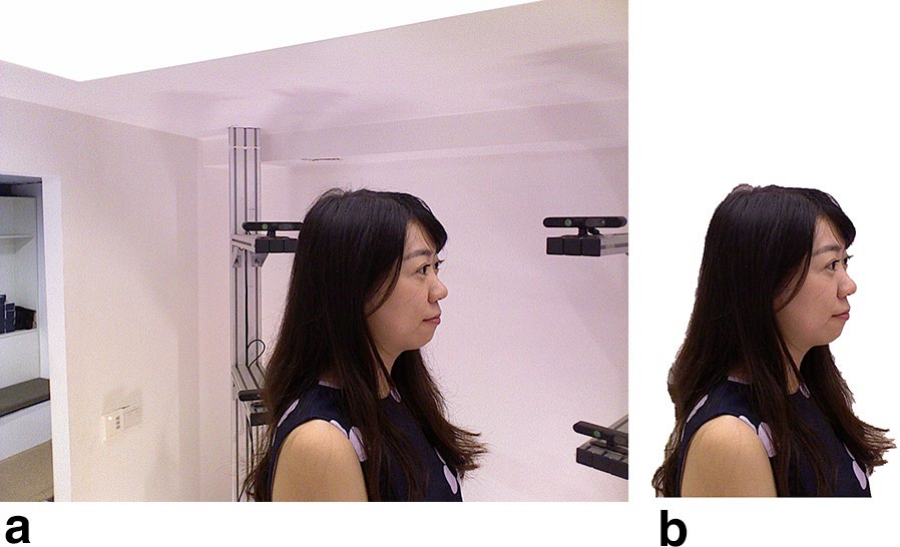
Images enhancement. *Left* is the original color image and *right* is the resulting color image

As a result, the background, irrelevant environment content, is removed out of the color images (Fig. 2).

### Seamless optimization

**Fig. 3:**
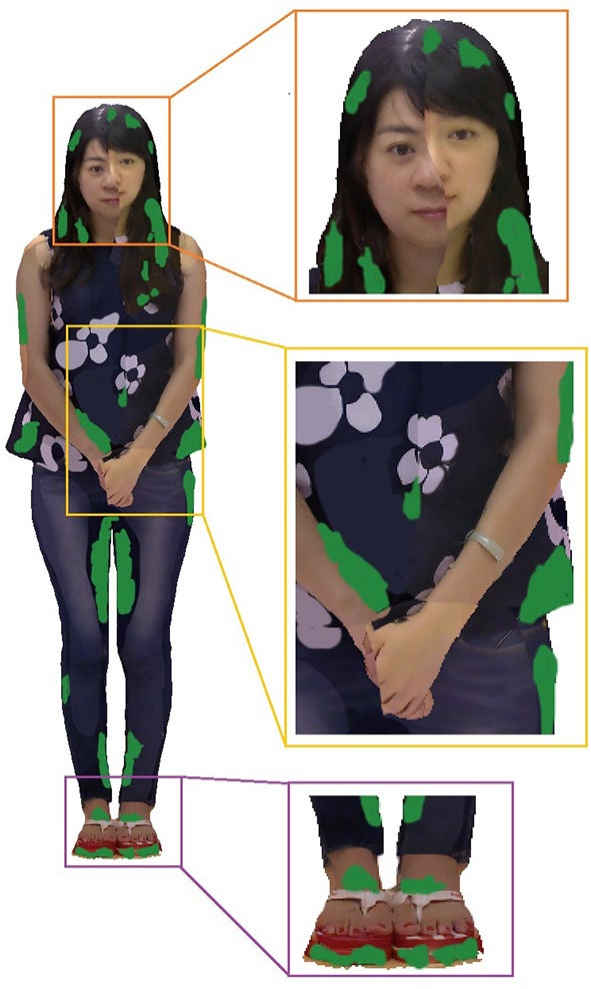
Initial texture on the surface of the woman model. Some misalignment seams, noticeable color diversity and blank regions without texture contents (*green part*) exist on the texture

The filtered color images from these cameras, which covers part of model respectively, should be stitched together to create an integrated texture on the model’s surface. Due to the multi-view misregistration and inaccuracy of depth data, it produces some undesirable artifacts in the initial texture, such as misaligned seams at the boundary between different color images, illumination variation between different parts of the texture, and blank regions without any texture contents. These artifacts are small but noticeable, and degrade the visual quality significantly, as shows in Fig. 3.

To eliminate these artifacts, we treat texture projection as an optimization of images stitching. Each triangular mesh of the model’s surface is projected onto the set of color images $\{I_1,\ldots ,I_N\}$, from which it is visible or not. We seek for the best color image with high contrast, low anisotropy and high resolution as its texture content, and then generate a seamless and smooth texture between adjacent triangular meshes. Actually it results in a Markov Random Field problem.

#### Markov Random Field model

It is a hybrid combinatorial continuous optimization to search a texture label *x* for each triangular mesh, which identifies its best associated color image. Then we create the Markov Random Field model as the following form.

7 $$\begin{aligned} \min _{I_1,\ldots ,I_N}\sum _{i=1}^N D_i(x_i) + \lambda \sum _{\{i,j\}\in M} V_{ij}(x_i,x_j) \end{aligned}$$

In the first term,

8 $$\begin{aligned} D_i(x_i) = - \delta _i \int _{\Phi (p)} \left\| \Delta \Phi _i (p) \right\| ^2 dp \end{aligned}$$

it calculates the data cost of each triangular mesh by projecting them onto each color image, where parameter $\delta$ is the ratio of triangle perimeter and area, and $\Phi (\cdot )$ is the projection operator from triangular mesh to color image *I*. Then we get the best texture label for each triangular mesh, and project the corresponding color image onto the triangular mesh , from which it is visible.

In the second term,

9 $$\begin{aligned} V_{ij} (x_i,x_j) = \int _{E_{ij}} \left\| \Phi _i(p) - \Phi _j(p) \right\| ^2dp \end{aligned}$$

the $E_ij$ means the same edge shared by the two adjacent triangular meshes, so it only calculates the smooth cost of texture discontinuities between the adjacent triangular meshes, and searches the image contents whose details are coherent at the shared edge. If both of the adjacent meshes have the same texture label, the term’s value equals zero. Therefore, the optimization of this term is mainly concentrated at the adjacent triangular meshes with different texture labels.

In this Markov Random Field model, each node denotes a triangular mesh with a set of optional texture labels, and the solution of the optimization substantially assigns a best texture label, namely color image, to each triangular mesh of the surface.

#### Optimization with adaptive iterative factor

To solve the optimization, we use the method based on graph cut, named $\alpha$-expansion, which refers to the min-cut/max-flow algorithms of graph theory into the combinatorial optimization. The minimal cut corresponds to the global minimization of the energy function, which assigns a best label to each triangular mesh within a global minimum. Many other standard algorithms, such as iterative conditional model (ICM) and simulated annealing (SA), apply small moves which just change one node’s label at one time. In contrast, the $\alpha$-expansion algorithm is allowed to change the labels of a large number of nodes simultaneously, aiming to obtain a smaller energy. Therefore, within such large moves, it is effective to get rid of the local minimum and rapidly converge to the global minimum.

**Fig. 4:**
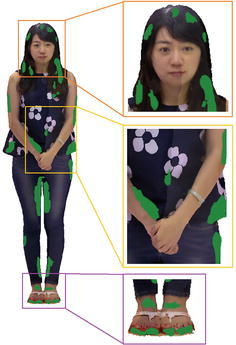
The texture after process of seamless optimization

The $\alpha$-expansion algorithm is summarized as follows. Given an arbitrary initial labeling *f* and a label $\alpha$, a weighted edge is formed between neighboring nodes, and the goal is to find a new labeling $f'$ which minimizes the energy over all the nodes’ labels through one $\alpha$-expansion move. During the $\alpha$-expansion move, it just changes the nodes whose label is not $\alpha$, and the other nodes whose label is $\alpha$ maintain the same value. If the energy of new labeling $f'$ is less than the original, it continues to perform another one $\alpha$-expansion move; otherwise, returns the current labeling $f'$ with the minimal energy. Therefore, the returned labeling $f'$ is the final solution of the energy function, which assigns a best label to each triangular mesh.

However, the $\alpha$-expansion algorithm can not eliminate the misalignment seams at the boundary between different color images, caused by multiple-view misregistration and inaccuracy of depth data. To minimize these artifacts, we introduce an adaptive iterative factor, a translation coordinate of the color image, to expand the texture label *x* as the form of $x=(I_i,t)$, where $I_i\in \{I_1,\ldots ,I_N\}$. Then, during the $\alpha$-expansion move, we can move the pixels in the color space of images adaptively and iteratively, to match the color content precisely at the edge shared by adjacent triangular meshes. As a result, the misalignment seams would be removed significantly. So the second term becomes this form

10 $$\begin{aligned} V_{ij} (x_i,x_j)= & {} \int _{E_{ij}} \left\| \Phi _i(p)\omega (t_i) - \Phi _j(p)\omega (t_j) \right\| ^2dp \end{aligned}$$

11 $$\omega (t)= \hat{\omega } (t - \Delta T) + e(t)$$

where $\omega (t)$ is the iterative function of the translation coordinate, and *e*(*t*) is the unit coordinate vector.

In the optimization of seams, we treat each color image as an individual image space. After each triangular mesh of the model’s face is projected on the color image, the location of the mesh on the color image is obtained. Then, with the adaptive iterative factor, a 2D translation vector, the location of mesh is changed along the possible directions. Finally, we get the best location by comparing the color content of two adjacent triangular meshes at the shared edge. In the initialization of the optimization, every triangular mesh is assigned a set of possible labels, that means the mesh can be projected onto these color images, and the adaptive factor is set to be zero. Then we calculate the energy function iteratively to find the optimal labels, and move the texture image gradually, with a threshold of 32 pixel units, to match the color content at the shared edge of adjacent meshes. Therefore, the misaligned seams, caused by the inaccuracy of depth data and multi-view misregistration, are eliminated. In each cycle of iteration, each node is assigned the current best label by energy optimization. Furthermore, to confirm the assigned label *x* being the best label for node *i*, we fix the neighbor nodes with their assigned best label, and calculate the sum of energy of $\sum V_{ij}(x_i^*,x_j)$ for all the possible labels of node *i*. If another label $x'$ achieves the minimal energy, we abandon the assigned label *x* and choose the label $x'$ as the best label for node *i*. As the iterative strategy is applied to the energy optimization, the result of current iteration is further fed into next iteration as candidate labels, until a best labeling $f'$ achieves the global minimum of the energy optimization over all the nodes’ labels. Finally, the optimized texture without misalignment seams is obtained, as Fig. 4 shows.

### Color blending

Although these misaligned seams can be eliminated effectively by the optimization of MRF, one artifact of illumination variation in different images still remains, caused by the different ray gains of RGB-Depth cameras from respective positions, as well as the reflection of surface. This small but noticeable artifact significantly degrades the texture and visual effect.

To resolve this artifact, we deal with color image as sources of color gradients rather than sources of color, and construct a composite vector field. Then we figure out the fused color of multiple images whose gradient best matches the vector field, and thus the texture between different parts of surface becomes coherent in illumination.

**Fig. 5:**
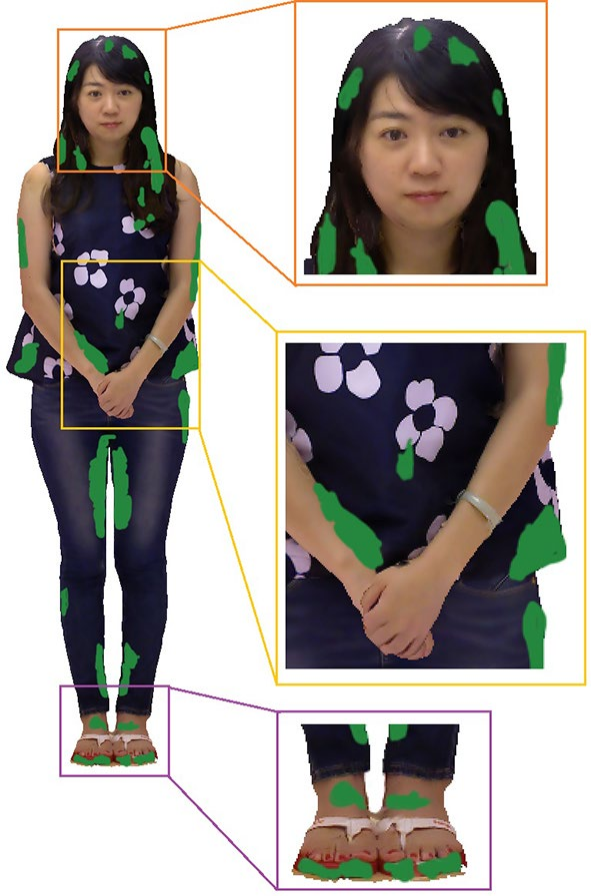
The result after applying color blending in gradient domain. s

As is well known, the color image consists of RGB three channels, so the process of color blending is performed separately in these three channels. In this case, given a single color channel, we refer to the color space of images as $D(D \in \mathfrak {R}^2)$, the color gradient as *g*, the composite vector field as *V*, and the boundary between adjacent color images as *b*, where the pixels keep fixed as constraints for color blending. Accordingly, the objective function is

12 $$\begin{aligned} \min _g \iint _{D} \left| \nabla f - \vec {V} \right| ^2,\quad f|_{\partial D} = g|_{\partial D} \end{aligned}$$

where gradient operator is $\nabla = [\frac{\partial }{\partial x}, \frac{\partial }{\partial y}]$.

The minimization of Eq. (12) could be transformed to the corresponding Poisson equation with Dirichlet boundary condition,

13 $$\begin{aligned} \triangle f = div\vec {V}, \quad f|_{\partial D} = g|_{\partial D} \end{aligned}$$

where the Laplace operator $\triangle = \frac{\partial ^2}{\partial x^2} + \frac{\partial ^2}{\partial y^2}$, and the divergence operator of the vector field $\vec {V} = (u,v)$ is $div\vec {V} = \frac{\partial u}{\partial x} + \frac{\partial v}{\partial y}$.

In the color space of images, the pixels are discretely distributed with the value *I*(*u*, *v*). Then the divergence of vector field $\vec {V}$ specifies two linear equations, each involves two variables,

14 $$\begin{aligned} I(u+1,v) - I(u,v)&= I_x (u,v)\nonumber \\ I(u,v+1) - I(u,v)&= I_y (u,v) \end{aligned}$$

which leads to a discrete Poisson equation. Besides, the fixed pixels in the boundary between adjacent images are added into the linear system as constraints. Consequently, the resulting linear system of Poisson equation is over-constrained. We use the method of multi-grid (Fattal et al. 2002) to solve the equation, and achieve the optimal gradient which best matches this vector field. As a result, each pixel in color images is assigned the best value. Then the color of different images becomes coherent without illumination variation (Fig. 5).

### Blank region repair

After the illumination variations are handled effectively by the process of color blending, last remaining artifact is blank regions, which is caused by inaccuracy of depth data and multi-view registration. As there is no corresponding relationship between the meshes in these areas and color images, some blank regions without any texture content exist on the surface of model, which destroy the integrity of texture.

**Fig. 6:**
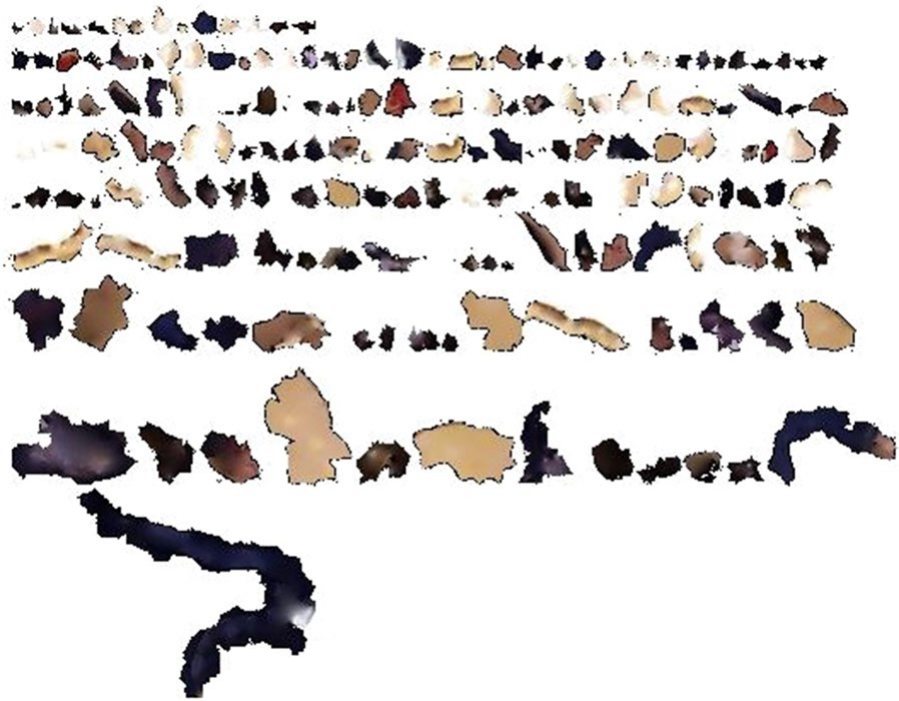
The generated texture image for blank regions

With the purpose of repairing these blank regions, we need to fill up these regions with color contents which are smoothly blending with neighboring regions, and generate an integrated and coherent texture on the model’s surface. In this paper, we apply the library of CGAL to parameterize the 3D textured surface (Lévy et al. 2002; Desbrun et al. 2002), and project it onto a 2D plane. Then we use the K-Nearest Neighbor algorithm to search for *K* neighboring points with color contents, and then create the texture image for blank regions with these color contents, shown in Fig. 6.

As a result,we project it onto the the model’s surface to fill up the blank regions, and then the entire surface of the model is textured. The process is interpreted as follows.

1. Search for *K* colored neighboring points of the blank point in the projected plane. While the number of colored neighboring points is less than *K*, the range of search extends to neighbors of these colored points until the number is equal or greater than *K*.
2. Calculate the distance respectively from the *K* colored points to the blank point, and refer to the maximum among them as Dmax. Given another colored point with distance *D*, compare it with colored point of $D_{max}$. If *D* is larger than $D_{max}$, then abandon the former; otherwise, replace the latter with the former of value *D*, and then update the maximum distance among the new *K* colored points.
3. Repeat step 1 and 2, until the *K* colored points no longer update.

**Fig. 7:**
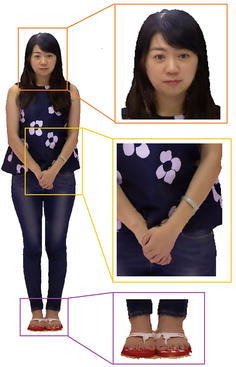
The result after repairing the blank regions

Then, a texture image for blank regions ia generated and back projected onto the model’s surface. Consequently all blank regions on the surface of model become textured with color contents, and an integrated and coherent texture is generated on the surface of model as Fig. 7 shows.

## Implementation and results

All the procedures of our method have been programmed on the platform of Visual Studio 2010 with Open GL. We implement and verify our method on commodity computer equipped with an Inter Xeon E5-2643 CPU of 3.3 GHz, 12GB RAM and NVIDIA Quadro K4000 graphics card.

**Table 1:** Energy and runtime of optimization for various methods

Models	Measure item	BP	Mosaicing	Montage	Ours
Woman	Energy	56,137,613	11,212,098	10,174,383	10,174,183
	Runtime(s)	104.64	10.68	1051.97	14.83
Man	Energy	64,274,859	13,793,729	14,706,724	11,474,016
	Runtime(s)	127.61	13.42	1166.19	20.16
Doll	Energy	346,328,438	8,148,734	7,633,248	7,633,137
	Runtime(s)	92.85	10.76	947.82	12.47

**Fig. 8:**
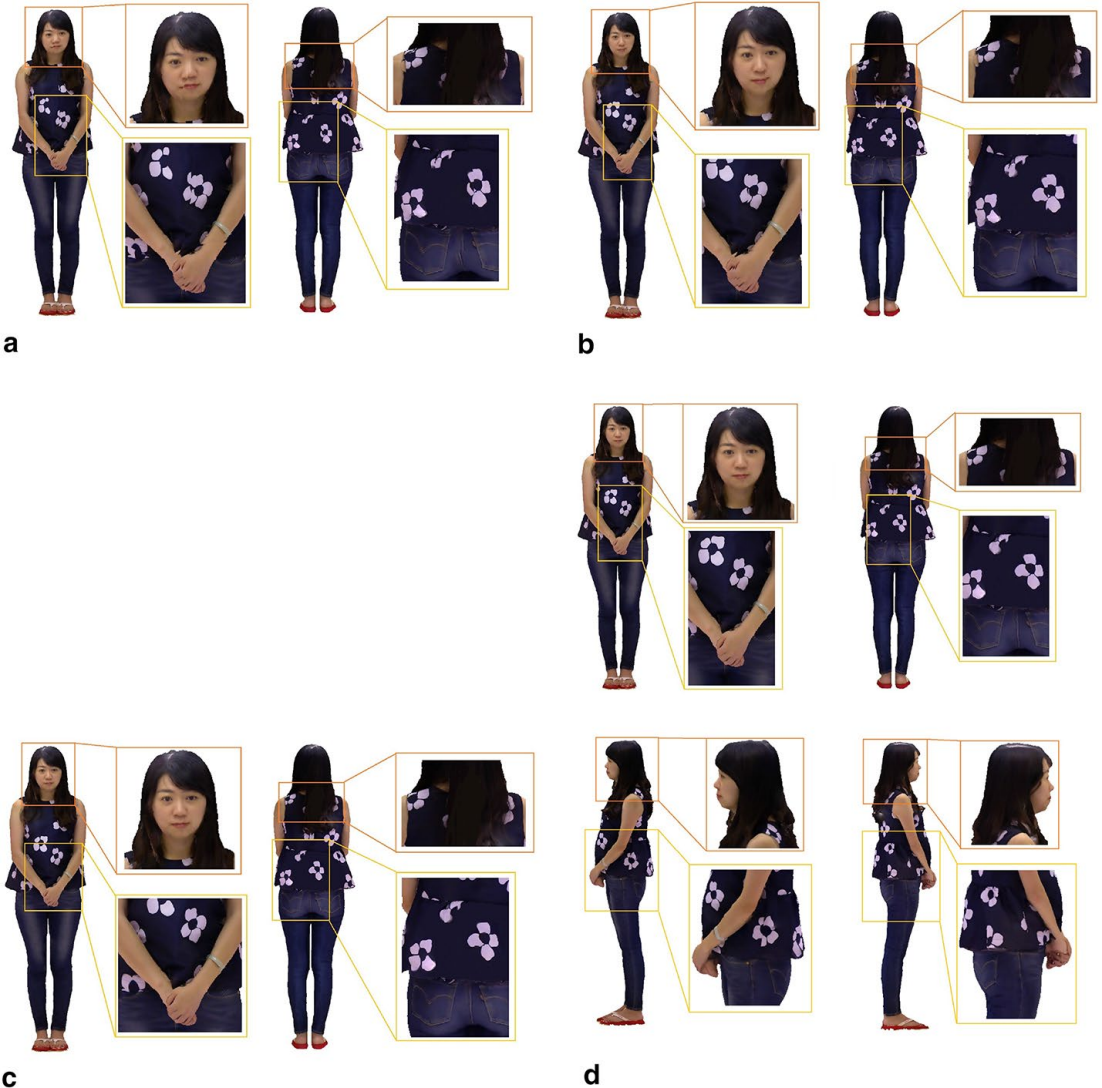
The final textures on the surface of the woman model generated by the four methods. **a** BP; **b** mosaicing; **c** montage; **d** ours from four directions

**Fig. 9:**
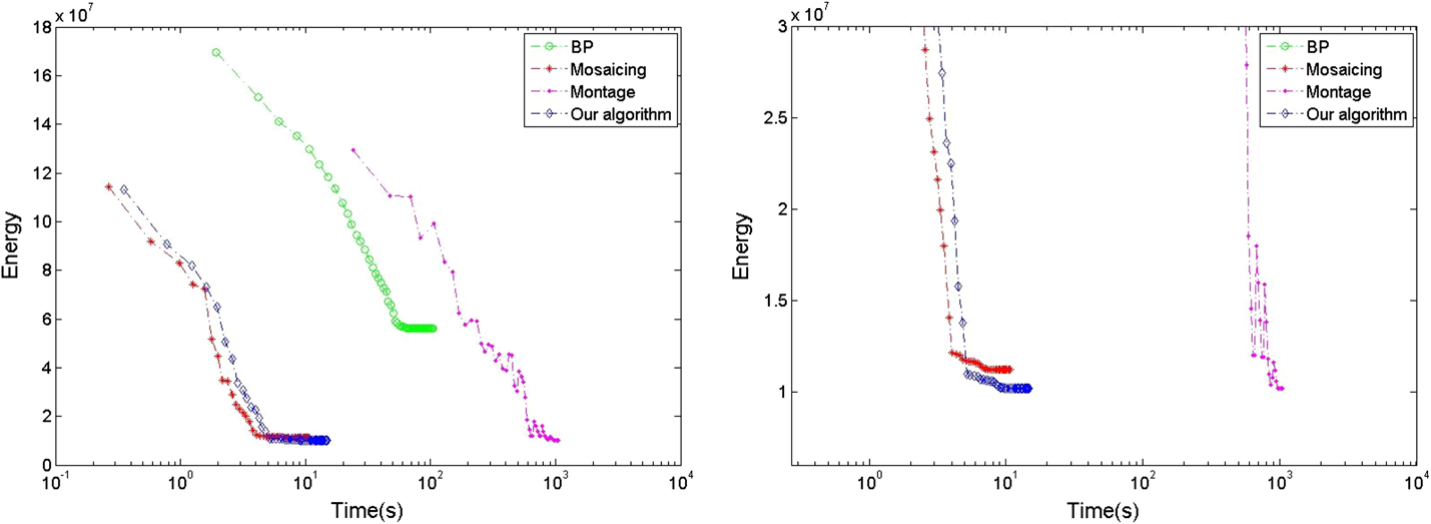
Results of energy minimization on the woman model. The *two plots* show the runtime on the *x*-axis using a log scale and the energy on the *y*-axis. Both of them show the same curves, and the *right one* is zoomed in on the *y*-axis to show the minimum energy much clearly, which may not contain the larger values. The plots in Fig. 11 are generated in the same manner

**Fig. 10:**
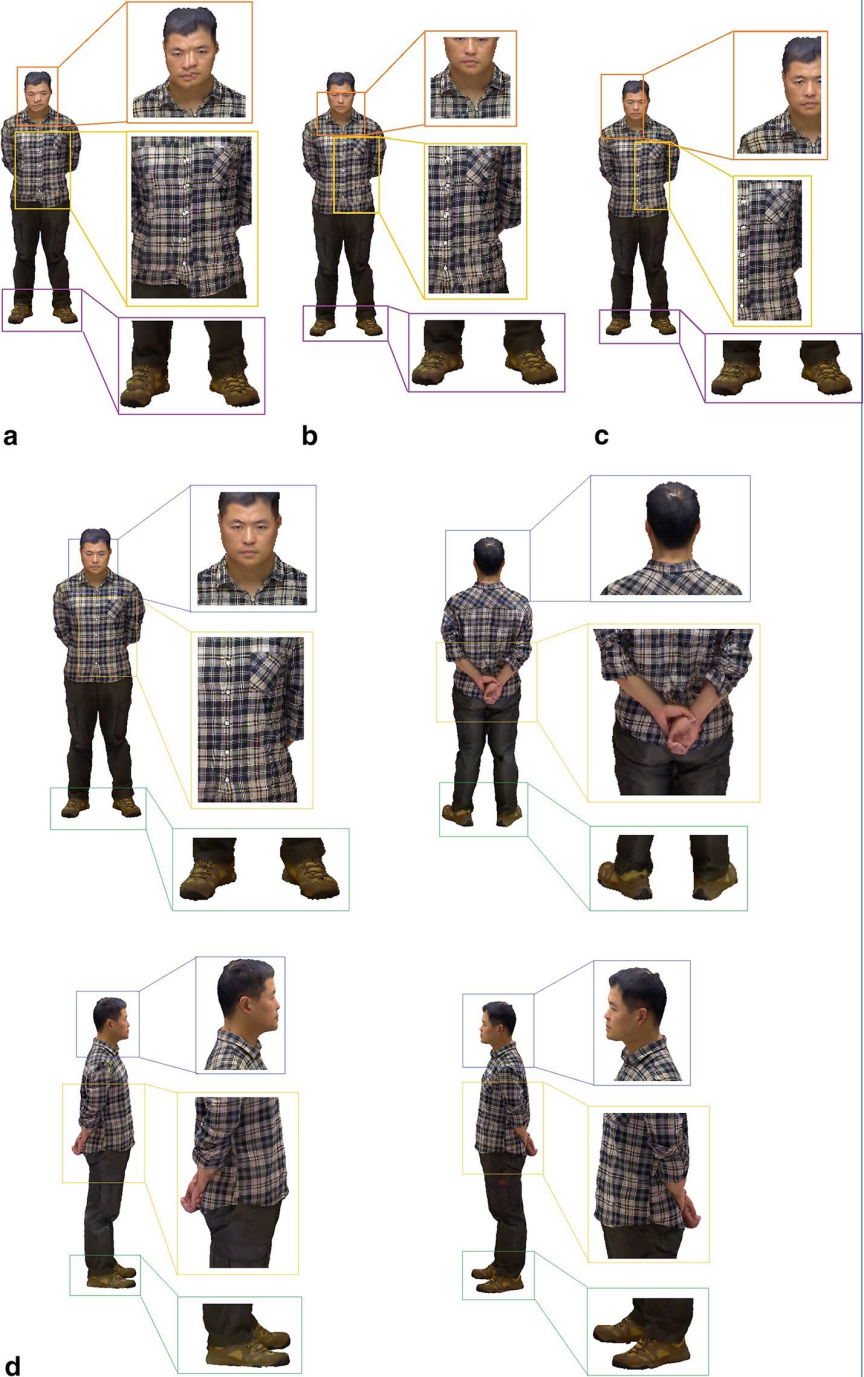
The final textures on the surface of the man model generated by the four methods. **a** BP; **b** mosaicing; **c** montage; **d** ours from four directions

**Fig. 11:**
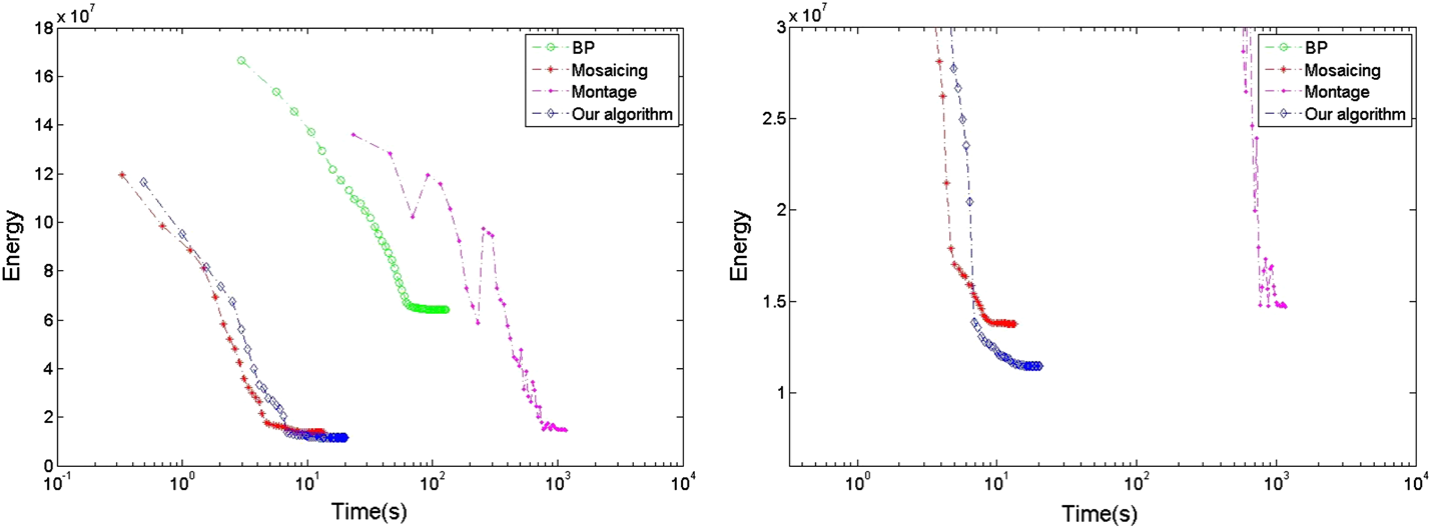
Results of energy minimization on the man model

To evaluate the effectiveness of our method, we perform our method and other three state-of-the-art methods on two human models and a doll model. The results of texture on model’s surface are shown in Figs. 8 and 10 respectively, the corresponding results of energy minimization are shown in Figs. 9 and 11, where the *x* axis shows the runtimes in seconds on a log scale and the *y* axis shows the energy of the different methods over time, and the energy and runtime of minimization are shown in Table 1. The other three state-of-the-art methods we compared are Belief Propagation(BP), Mosaicing(using graph cuts), and Montage(using Tree-Reweighted Message Passing, namely TRW-s).

In Fig. 8, we can see that, the texture generated by BP algorithm is much worse than other methods. There are obvious misaligned seams at the boundary between adjacent color images, and some images ghost as well as incorrect details exist in the part of face, hand, jacket and trouser pocket. These artifacts degrade the quality of texture and visual effect significantly. Although the method of Mosaicing achieves a better result than BP, it can not handle with the seams of misalignment and images ghost, which exists in the part of lip, jacket details, hands and so on. Both the methods of Montage and ours generate the best texture on the surface of the model. They remove out these artifacts, such as seams of misalignment, images ghost and incorrect details, and create a seamless, integrated and smooth texture on the surface of model.

In the corresponding result of energy minimization (Fig. 9), the plot shows the energy of different methods over time and some interesting features. The BP algorithm just converges to a local minimum, far away from the global minimum, and the runtime is at the level of $10^2$ s. The Mosaicing method finds a near-optimal solution most quickly at the level of $10^1$ s. Although the energy of Montage gradually approaches to the global minimum, it takes a large amount of time at the level of $10^3$ s. Furthermore, during the process of calculation, it does not decrease progressively but oscillates widely. That means, if it is used in limit time and iterative frequency, it would not achieve the global optimal. Our method achieves the global minimum by aligning the texture contents at the boundary between adjacent color images, and converges quickly and progressively at the level of $10^1$ s, just slightly slower than the Mosaicing method.

Due to the alignment of texture at the boundary, the optimization of our algorithm is speeded up. With the adaptive factor, the texture of mesh could be moved along the direction, in which the energy of optimization decreases progressively and the adjacent meshes match the color content gradually at the shared edge. Besides, according to the picture of energy minimization, the alpha-expansion algorithm converges straightforward to the global minimum without any oscillation, which reduces the time expenses. As the Fig. 9 shown, the energy of different methods demonstrates that our method obtains the minimum of energy, and achieves effectively enhancement in the aspect of runtime.

Given the second example of a man model (Figs. 10 and 11), similar to the result of the woman model, the BP algorithm gets the worst performance, which converges a local optimal, and can not deal with the previous artifacts. The Mosaicing method obtains a solution close to the global optimal most quickly, but can not remove out the seams of misalignment and images ghost. Our method obtains the global minimum of energy optimization quickly, just a little slower than the Mosaicing method, and creates the best texture on the surface of the man model. In contrast, The result of Montage seems to present something interesting and different. There are some seams of misalignment and images ghost in the region of lip and shirt, presumably caused by its drastic oscillation in the process of energy minimization. According to the result of energy minimization (Fig. 11), it demonstrates that the Montage method oscillates widely and doesn’t achieve the global minimum at the end of optimization.

**Fig. 12:**
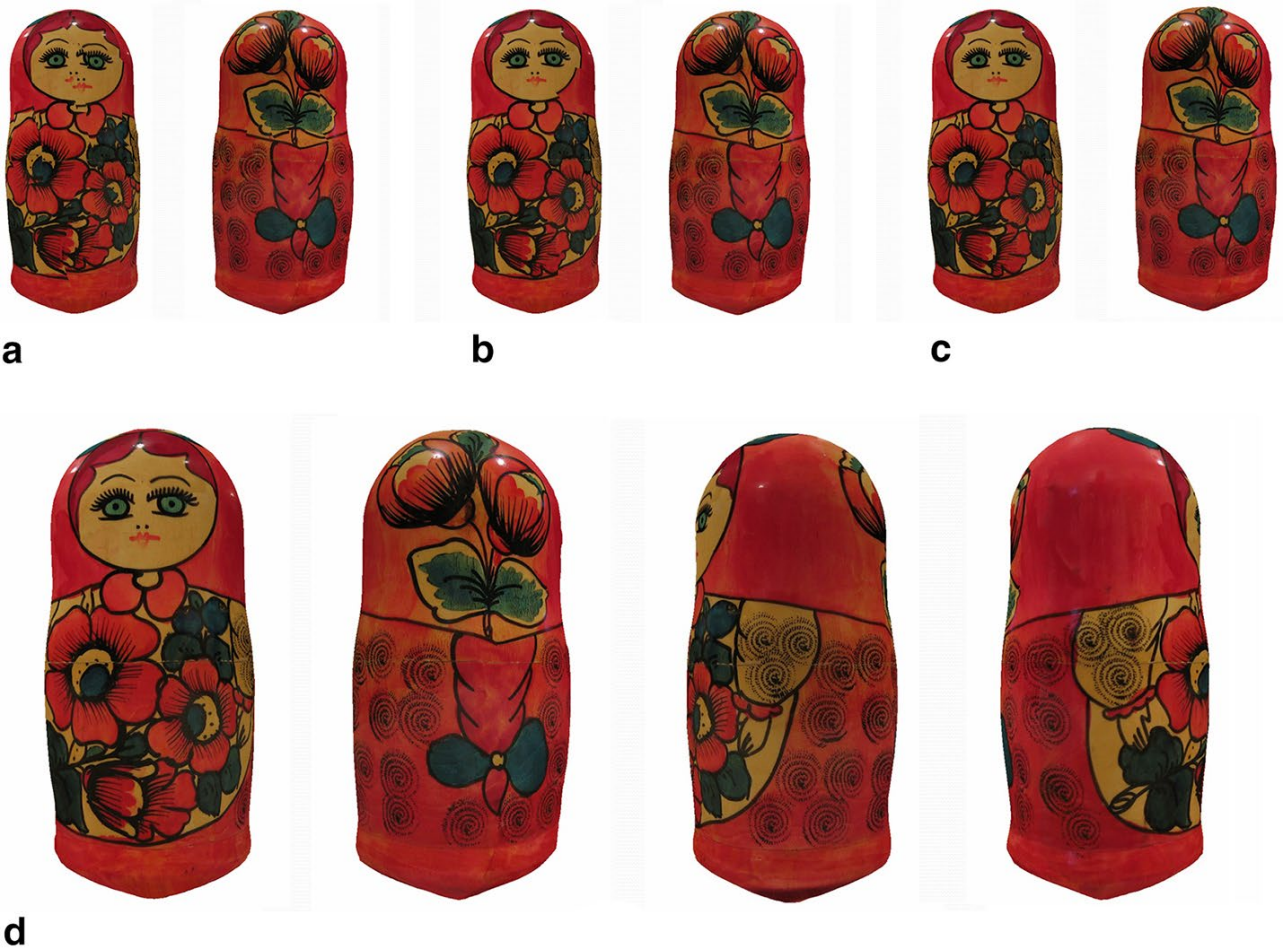
The final textures on the surface of the doll model generated by the four methods. **a** BP; **b** mosaicing; **c** montage; **d** ours from four directions

Furthermore, to evaluate the effectiveness of our method, we implement these methods on the doll model, which is previously published in Mosaicing and Montage. As shown in Fig. 12, some noticeable seams and ghost artifacts are produced on the result of BP, as BP algorithm converges to a local minimum and could not align the adjacent meshes at their shared edge. Obviously, these undesirable artifacts seriously degrade the performance of BP. Although superior to the BP, the Mosaicing can’t eliminate the seams. Due to the error of multi-view registration and inaccuracy of depth data, the adjacent meshes can not match their color content at the shared edge without moving the texture image. Thus the seams and broken texture are still noticeable. In contrast, the Montage brings out a desirable texture in visual observation, without any seams and ghost artifacts. But it needs sufficient iterations to converge to the global minimum, which takes a lot of time. Otherwise, if the number of iteration is insufficient, the performance of Montage will severely decline, especially when it is in the oscillation, just as the result of the man model. Meanwhile, our method also provides a seamless texture. In the process of optimization, the misaligned textures are moved with the adaptive factor to match their color content at the shared edges, and the energy decreases progressively and converges straightforward to the global minimum without any oscillation. Thus, our method achieves the optimal result much faster than Montage, as listed in Table 1. Based on the experiments of our human models and the previously published doll model, the comparison indicates that our method is effective for reduction of seams, with a rapid optimization.

According to the experiments, we know that the BP algorithm may go into a region of local minimum and could not step out to converge to the global minimum, and some misaligned seams and distortions still remain at the shared edge of adjacent meshes through the Mosaicing algorithm. Although the Montage algorithm generates a favorable texture in visual observation without any seams, it needs abundant iterations and larger time expense to converge to the global minimum, especially accompanied with wide oscillations. In order to create a seamless texture on the model’s surface with a rapid optimization, we propose a method of introducing an iterative factor into the $\alpha$-expansion algorithm. As the $\alpha$-expansion algorithm is able to change the label of several nodes simultaneously, with such a large move, it is effective to get rid of the local minimum and converge rapidly to the global minimum during the optimization. Furthermore, with the iterative factor, the texture of mesh could be moved gradually to match the color contents at the shared edge of adjacent meshes, which compensates for the inaccuracy of depth data and multi-view misregistration. As the texture is aligned at the boundary, the energy of optimization decreases dramatically and converges straight towards the global minimum without any oscillation, leading to a large acceleration of optimization. However, the major limitation of our proposed method is model’s movement. That means, the person should keep motionless as a statue until the color image and depth data have been obtained by the cameras. If the person moves his or her body during the process, the movement will result in terrible damage to the corresponding relationship between color images and depth data, and the error of mismatch is so huge that our method could not handle effectively. In future work, we will take the slight movement of human body into consideration, which involves the nonrigid registration of human body.

## Conclusion

In this paper, we present an effective method to generate a seamless, integrated and smooth texture on the surface of 3D human models. Given a set of color images obtained by multiple RGB-Depth cameras around the human model, and a surface mesh reconstructed previously by point cloud, we establish a MRF model. To eliminate the seams at the boundary between adjacent color images, caused by multi-view misregistration and inaccuracy of depth data, we introduce an adaptive iterative factor into the MRF model. To smooth out the noticeable illumination variation between different color images, we apply the state-of-the-art method of Poisson blending to a composite vector field in gradient domain. Furthermore, we perform parameterization of the model’s surface and apply KNN algorithm to repair the blank regions without texture content. To evaluate the effectiveness and robustness of our method, we compare it with another three advanced methods. The results demonstrate that our method achieves the global minimum of optimization almost fastest and creates a best texture on the surface of models. Therefore, our method proves evidently effective and preferable both in visual effect and quantitative analysis.
